# Supplementary material for: The Biological Origin of Linguistic Diversity
Source: PLoS One. 2012 Oct 30;7(10):e48029. doi: 10.1371/journal.pone.0048029 (PMC3484145; doi:10.1371/journal.pone.0048029)
Supplement: Information S1 — Contains the derivation of Equation 1 . (DOC) [file pone.0048029.s001.doc]

Electronic Supporting Information S1

**The Biological Origin of Linguistic Diversity**

*Andrea Baronchelli^1^, Nick Chater^2^, Romualdo Pastor-Satorras^3^*,

and *Morten H. Christiansen^4,5,*^*

^1^ Laboratory for the Modeling of Biological and Socio-technical Systems,

Northeastern University, Boston, MA, USA

^2^ Behavioural Science Group, Warwick Business School,

University of Warwick, Coventry, UK

^3^ Departament de Física i Enginyeria Nuclear,

Universitat Politècnica de Catalunya, Barcelona, Spain

^4^ Department of Psychology, Cornell University, Ithaca, NY, USA

^5^ Santa Fe Institute, Santa Fe, NM, USA

^*^ Morten H. Christiansen, E-mail: *christiansen@cornell.edu*

**Derivation of Equation 1**

Consider two languages, *A* and *B,* and the *i*th linguistic principle, *L_i_*. Given that the principle is a binary variable, either the two languages share the same value of the principle (i.e., they both have *+L_i_* or *-L_i_*) or they are misaligned (one of them has *+L_i_*, and the other has *–L_i_*). In the first case, we say they are in *s* state, and in the latter that they are in *d* state. The probability of a switch from the *s* state to the *d* state is twice the language mutation rate *l*, since it is produced every time one of the two languages mutates. Calling *P(x)* the probability of having the two languages in state *x*, and noting that *P(d)=1-P(s)*, we have:

$\frac{\partial P(d)}{\partial t}=2l[P(s)-P(d)]=2l[1-2P(d)]$.

The Hamming distance between the two languages and on the given principle is *H(A,B)=1* if the two languages are entirely in the *d* state; *H(A,B)=0* when they are entirely in the *s* state; and the average value of H(A,B) is exactly *P(d).* Considering that right after a population split, the two languages are in the *s* state, the above equation straightforwardly yields the solution:

$P(d)=H(A,B)=\frac{1-e^{-4lt}}{2}$,

which also describes the time evolution of *D_L_(A, B)*, because linguistic principles evolve independently. It is worth noting that this argument relies on the assumed independence of the different linguistic principles. An alternate scenario in which some principles are correlated with other is beyond the scope of the present paper, but likely to be worth future investigations.
